# 

**Published:** 1887-11

**Authors:** 


### CONTENTS.

ORIGINAL COMMUNICATIONS—

The Early Diagnosis of Spinal Caries, with Remarks on Treatment. By H. P. Cooper, M. D., Atlanta, Ga. .......... 517

Atheroma of the Left Coronary Artery, Resulting in Aneurism of the Apex of the Left Ventricle. By Robert Tilley, M. D., Chicago, Ill .......... 526

CLINIC REPORTS—

Report of Two Cases of Fracture. By F. M. Ridley, M. D., La-Grange, Ga .......... 531

SOCIETY REPORTS—

Chicago Medical Society .......... 534

CORRESPONDENCE—

Our New York Letter .......... 542

Twins—An Interesting Case .......... 545

Laceration of the Perineum .......... 550

BOOK REVIEWS—

Lessons in Gynæcology. By William Goodell, A. M., M. D .......... 552

Gray's Anatomy .......... 554

The Diagnosis and Treatment of Hæmorrhoids. By C. B. Kelsey, M. D .......... 554

EDITORIAL—

Post-Graduate Study .......... 556

Annual Opening .......... 557

A Fraud Exposed .......... 559

A Doctor's Shop of the Olden Time .......... 561

Items .......... 562

